# Estimation of chronic dietary intake of pesticide residues

**DOI:** 10.11606/s1518-8787.2021055002197

**Published:** 2021-06-17

**Authors:** Jacqueline Mary Gerage Marques, Marina Vieira da Silva

**Affiliations:** I Universidade de São Paulo Escola Superior de Agricultura Luiz de Queiroz Programa de Pós-Graduação em ciência e tecnologia de alimentos PiracicabaSP Brasil Universidade de São Paulo. Escola Superior de Agricultura Luiz de Queiroz. Programa de Pós-Graduação em ciência e tecnologia de alimentos. Piracicaba, SP, Brasil; II Universidade de São Paulo Escola Superior de Agricultura Luiz de Queiroz Departamento de Agroindústria, Alimentos e Nutrição PiracicabaSP Brasil Universidade de São Paulo. Escola Superior de Agricultura Luiz de Queiroz. Departamento de Agroindústria, Alimentos e Nutrição. Piracicaba, SP, Brasil

**Keywords:** Food Pollutants, Chemical, Agrochemicals, adverse effects, Agro Toxic Maximum Allowable Limit on Food, Pesticide Exposure, Food and Nutrition Security

## Abstract

**OBJECTIVE:**

To estimate the maximum theoretical daily intake of pesticides potentially consumed, chronically, by the Brazilian population.

**METHOD:**

By using data from the food consumption section of the 2008–2009 Household Budget Survey to characterize the population diet, a database was built to group the foods based on the NOVA classification. Considering the maximum residue limit values of each pesticide authorized in the country until 2016, the limits of all consumed foods were added and multiplied by the amount consumed, resulting in the maximum theoretical intake index, which was compared with the acceptable daily intake.

**RESULTS:**

The results show that, of the 283 pesticides considered in the database, 71 (25%) compounds had estimates of zero intake, 144 compounds (50.8%) reached acceptable daily intake values and 68 compounds (24%) showed median intake that exceeded the acceptable daily value. The pesticide intake estimation according to the different regions of the country showed a variation in the amount of compounds that exceeded the acceptable daily intake (48 to 69 substances) due to the different consumption patterns. The categories of products that most exceeded the limits were the insecticides, herbicides and fungicides.

**CONCLUSION:**

The application of this methodology is valid for the first step in risk assessment, but the resulting values may be different from the actual exposure since they do not include other factors, such as the combined use of pesticides or unauthorized products. The importance of developing research on specific national food consumption data in a systematic way is emphasized, which generates data and analyses that allow a detailed risk assessment.

## INTRODUCTION

The relation between food supply and consumption by the population, considering both availability and access, is the basis for food and nutritional security. The subject involves different areas of knowledge, such as health, agriculture, economy, food and environment, reaching different meanings. The concept of food security is evolving, being also related to sustainability, which has had an increasing importance in a scenario of climate change, affecting prices and causing interruptions in the food supply chain. Nutrition and food sovereignty also contribute to the improvement of this concept^1^.

The large-scale production of commodities in Brazil, driven by the increased global demand for food, increased by 135% the total consumption of pesticides from 2000 to 2014^6^, thus enhancing the exposure to their residues. Pesticide residues can be either a substance or a mixture of substances remaining in food or in environment. Besides, they can also be the conversion, degradation, and reaction products, and metabolites and impurities with toxicological importance^7^. Acute poisoning occurs due to exposure to large amounts of residues, and the chronic form due to a long-term exposure to small amounts. Not only fresh plant-based foods show toxicological potential, but also prepared, such as homemade recipes, and processed foods, found in any supermarket.

Few studies assess the risks of pesticides poisoning from food consumption, especially for the Brazilian population. This assessment is based on the identification of potentially hazardous substances, thus allowing risk characterization. Population consumption data and anthropometric data contribute to the development of this type of assessment.

Our study aimed at estimating the maximum theoretical daily intake (TMDI) of pesticides potentially consumed by the Brazilian population in food, based on consumption data obtained in the Household Budget Survey (HBS) conducted by the Brazilian Institute of Geography and Statistics (IBGE) between 2008–2009, emphasizing the substances that exceeded the intake limits, considering the products authorized for use in Brazil until 2016.

## METHODS

The database used in the estimation was built using the microdata of the food consumption section of the HBS, which records the consumption of the individuals aged 10 years or older. Data on a sample of 13,569 households were available, of which 33,613 individuals whose anthropometric data were fully identified were considered eligible, since the proposed analyses required the information on each individual’s body weight.

In the food consumption section of this survey, food items are recorded according to their consumption form. In the construction of the database, the foods were grouped according to the respective agricultural crops, and then the data on residue limit found in the monographs of each pesticides were applied^8^. Using the NOVA classification^9,10^ as a basis, the food was classified into three groups: fresh, processed, and prepared.

Fresh foods were considered those that do not receive any kind of preparation. Prepared foods involve several ingredients, which were identified in proportion, based on standard recipes and reference tables^11^. Regarding processed foods, the composition of food as a percentage of ingredients was researched, based on product labels, current laws of the National Health Surveillance Agency (Anvisa) and/or of Ministry of Agriculture, Livestock and Supply^15^, reference tables^11^ and internet search.

Foods of animal origin were also included in the database, which were grouped into: beef, pork, chicken meat, poultry meat, sheep meat, goat meat, mammalian meat, cattle offal, poultry offal, eggs and milk. For these groups, we used the maximum residue limit (MRL) values obtained from the *Codex Alimentarius*^23^, considering only the compounds authorized for use in Brazil, since Anvisa has no guidelines covering this category of products on the subject.

Food and beverages from the light, diet and organic categories, and foods whose composition could not be found or lacked MRL value defined by Anvisa were excluded from the study. At the end, a total of 743 foods were selected to data analysis.

The MRL values recorded in the monographs of pesticides (n = 283) with authorized use in the country until 2016 were adopted. The MRL values were summed according to the record of food consumed obtained from the HBS and multiplied by the amount consumed, resulting in the TMDI, which indicates the degree of exposure, according to Equation 1.

**Equation 1.** TMDI estimation^a^.

$$TMDI=\sum \left({MRL}_{i}\times F_{i}\right)$$

The results were compared with the values of acceptable daily intake (ADI), published in the pesticide monographs by Anvisa, according to Equation 2. When the national data were not existent, the values of international agencies such as the Environmental Protection Agency (EPA), *Codex Alimentarius* and the Australian government health department^23^ were used. The lowest value was used when there was more than one record for the same compound.

**Equation 2.** TMDI comparison with ADI.

$$\%ADI=\frac{TMDI\times 100}{ADI\times \text{ body weight }}$$

## RESULTS

Of the 283 pesticides studied, 71 compounds (25%) had an estimate of intake equal to zero. We also identified that other 144 compounds (50.8%) met the ADI values, and other 113 had a value recommended by Anvisa. Table 1 shows that the remaining 68 compounds (24%) had a median intake that exceeded the ADI value.

**Table 1:** Extrapolation intervals compared to acceptable daily intake (ADI).

Extrapolation intervals compared to ADI	Pesticide
(1~2) X > ADI (n = 32)	Acetamipride, alachlor, aldicarb, azoxystrobin, beta-cypermethrin, bifenthrin, captan, carbosulfan, cyproconazole, dithiocarbamates, edifenphos, Esfenvalerate, famoxadone, fenoxaprop, fenpropathrin, fluazifop-p, maleic hydrazide, iprodione, malation, mancozeb, molinate, novalurom, propargite, prothioconazole, chinometionate, sulfentrazone, tebenzurom, tetradione, thiamethoxam, thiophanate-methyl, tiram, triazofos.
(3~4) X > ADI (n = 18)	Cadusafos, carbendazim, clethodim, chlormequat, chlorpyrifos, dimethoate, epoxiconazole, etofenprox, fenamifos, phosmet, gamma-cyhalothrin, haloxifop-p, iminoctadine, mevinphos, MSMA, paraquat, propineb, protiophos.
(5~6) X > ADI (n = 6)	Carbaryl, carbofuran, deltamethrin, diafenthiurom, pirimiphos-methyl, tetraconazole.
(7~8) X > ADI (n = 2)	Dissulfoton, ethion.
(9~10) X > ADI (n = 3)	Diquat, diurom, propanil.
> 10 X ADI (n = 7)	Acephate, methyl bromide, diazinon, fentin, fipronil, phosphine, terbufos.

Table 2 indicates the 10 compounds with the highest estimates of intake by the population, of which seven (acephate, methyl bromide, diazinon, fentin, fipronil, phosphine and terbufos) exceeded ten times the ADI value. The other three (diquat, diurom and propanil) exceeded nine to ten times the ADI value.

**Table 2:** Estimation of pesticides most consumed by the Brazilian population through diet.

Pesticide	ADI (mg/kg)	Value of median consumption (mg/kg body weight)	Toxicological classification
Methyl bromide	0.0004 (AU)	1.527778	I - extremely toxic
Phosphine	0.0003 (EPA)	0.007711	II - highly toxic
Fipronil	0.0002	0.004866	II - highly toxic
Acephate	0.0012	0.022826	III - moderately toxic
Diazinon	0.002	0.030805	II - highly toxic
Phenine	0.0005	0.006956	II - highly toxic
Terbufos	0.0002	0.002632	II - highly toxic
Diquat	0.002	0.020833	II - highly toxic
Diurom	0.002 (EPA)	0.020669	III - moderately toxic
Propanil	0.005 (EPA)	0.045147	III - moderately toxic

ADI: acceptable daily intake; AU: Australian government; EPA: Environmental Protection Agency of the United States of America.

The pesticide intake estimation according to the different regions of the country showed a variation in the amount of compounds that exceeded the ADI due to the difference in consumption patterns and diversity of foods conditioned by different variables, especially family income, habits, cultural identity of the population and food regional availability. Table 3 indicates the amount of compounds that exceeded ADI by region.

**Table 3:** Amount of compounds that exceeded acceptable daily intake according to the region.

Region	Number of pesticides (compounds)
North	59
Northeast	62
Southeast	69
South	48
Midwest	69

In the North region, the same ten compounds with higher median intake obtained for the entire Brazilian population were observed. Methyl bromide compound remained as the most consumed, with different amounts of consumption of the other compounds. Propanil, a product with few records of poisoning and indicated for application in rice crop^26^, was the second most consumed.

The dissulfoton compound exceeded the ADI limit only in the Northeast region. This is an organophosphate insecticide applied in coffee culture, the second food item with the highest number of references in the food record of the first day of the HBS 2008–2009, which has the highest *per capita* consumption in the country in this region, reaching 230.4 g/day^26^.

For the Southeast, the ethion compound was identified among the ten compounds with the highest intake potential. This is a compound indicated for foliar application in pineapple, cotton, eggplant, coffee, citrus, apple, watermelon, melon, pear, pepper and tomato crops^26^, which are foods commonly consumed in the states of the region.

In a specific analysis, we used only data related to the state of São Paulo, since its socioeconomic characteristics differ from other states and regions, being used information related to 2,250 individuals. Ethion and propamocarb compounds exceeded ADI limits in this state. Studies show that residues from both compounds were reduced by processing procedures, such as oil refining, washing and cooking^29,30^. However, only food processing cannot guarantee they reach the ADI levels.

The South region stands out for presenting a smaller amount of compounds that exceeded the ADI limit. According to data from Vigitel^31^, the capitals of the states of the Southern region are among the ten cities considered the major consumers of fruits and vegetables in the country, being the best index identified in Florianópolis, state of Santa Catarina. Regarding the use of pesticides, the states with the highest consumption record are Paraná, with 11.6%, and Rio Grande do Sul, with an average consumption of 10.2% of the total applied in the country^32^. On the other hand, this was the region that recorded the highest consumption of organic foods, according to the HBS.

Regarding the Midwest region, the profile of the compounds that exceeded ADI limits was similar to that of the state of São Paulo, especially for the ethion compound.

## DISCUSSION

Chronic exposure to residues of organophosphate and carbamate pesticides, categories in which most pesticides exceeded ADI, can be related to a number of symptoms, such as neurotoxic effects, chromosomal alterations, liver and kidney damage, arrhythmias, allergies, asthma, Parkinson’s disease, different types of cancer and hearing loss^33^. The products in these categories can also act as acetylcholinesterase inhibitors, which affects the transmission of nerve impulses to the brain.

Methyl bromide, which stood out as the product with the highest intake potential, is applied in wooden crates for post-harvest storage. This compound has a high MRL, but a low ADI, which justifies the high value of TMDI, calling attention to the potential for high intake. In general, herbicides, one of the most commercialized categories of pesticides in the world, have high ADI values when compared with fungicides and insecticides, and therefore the category did not stand out in the results.

This type of evaluation is valid as a first step in risk assessment, since MRL is not the best indicator for estimating average residue levels. Likewise, performing the sum of all doses of pesticides is not ideal for assessing the cumulative risk, and the cumulative and synergistic effects of substances that cause the same toxic effects on tissues, organs and physiological systems and are able to produce joint and cumulative toxicity should also be studied – even if they have different modes of action^34^.

We emphasize that our estimates do not consider the volume of pesticides illegally acquired, which enter the country without permission, or even the use of counterfeit products, which are not as much efficient and safe as the products attested by the responsible agencies. Trade in illegal pesticides accounts for 24% of Brazilian agricultural pesticide market, according to data from the *Federação das Indústrias do Estado de São Paulo* (FIESP – Federation of Industries of the State of São Paulo)^38^.

An efficient program for monitoring the waste and the system of application of pesticides conducted continuously is essential as a strategy for the management of food safety in the country. Our results may support measures such as field education for the application of good agricultural practices, health risk assessment due to exposure and reassessment of products and substances.

We also would like to stress the use of individual food consumption data in the database. The proportional use of samples of population groups is important for this type of estimation, including stratification by age group, gender and education level.

The study by Meira et al.^39^ gathers estimates of pesticide consumption, focusing on the group of schoolchildren from the public school system of a municipality of the state of São Paulo. The authors show that of the 272 compounds considered in the study, 58 exceeded the maximum intake value. Some of these compounds may act as endocrine disruptors, which may lead to development changes, a fundamental concern for the sample in question.

This evidences the need to encourage the development of specific national studies on processing factors for the levels of residues found. The studies available show that all types of processing contribute to reduce risk due to exposure^40^–^47^

A survey conducted in a vegetarian community in Israel^48^identified an increase in the concentration of organophosphate residues in urine samples, and residents that consumed a larger portion of organic foods showed lower results for pesticide residues. Thus, the consumption of organic products can offer some protection against exposure. Agroecology is a form of sustainable management of agricultural production, considering social, political, cultural, environmental and ethical issues – working condition in the field, compatibility of culture to the ecosystem and the level of industrialization of the whole process. It is a modality that avoids the use of pesticides and chemical fertilizers and stimulates the planting of organics, being driven by family farming. However, the results of the production at scale, the costs involved and the time allocated to planting and harvesting, and productivity, should be considered.

The perspectives for future use of pesticides should be evaluated, not only regarding the effects on crops, occurrence of pests and pesticide efficiency, but also their implications for the technological development of regulations and economic situation. Given the expectations around climate change, the increase in temperatures will act on the change in rainfall regimes, favoring the development of pests and pathogens. Consequently, an increase in dosage, frequency of application and types of pesticides is expected. Therefore, new compounds may emerge, and both application of combinations and toxicity of the assets may increase, thus increasing the dietary intake of residues^49^.

Few studies have investigated the ecotoxicological effects resulting from the application of pesticides in tropical regions, and studies applied in temperate regions are mostly used as references. The adaptation of existing tests for tropical regions is indicated^47^.

## CONCLUSION

The guarantee of residue-free foods integrates the human right to adequate food, reinforcing the need for rigorous monitoring of pesticide residues, considered important for a national food and nutrition safety program. According to the results, many pesticides applied in crops consumed in Brazil have an intake potential that may exceed the recommended indices. Thus, we suggest that the national health system evaluate the real potential of exposure to pesticide residues, also due to perspectives of global climate changes, which would imply changes in the use of these products.

Systematic generation of food consumption data is also necessary to assess chronic, acute and cumulative risks more realistically. There is no way to assess the effects of exposure through the consumption of food and water, we can only use models based on the precautionary principle; however, it is already known that a low-dose exposure already induces cell death and reduces cell viability.

## Footnotes

a World Health Organization. Guidelines for predicting dietary intake of pesticide residues (revised): prepared by the Global Environment Monitoring System – Food Contamination Monitoring and Assessment Programme (GEMS/Food) in collaboration with the Codex Comitee on Pesticide Residues. Zurich, 1997, 40 p.
