# Supplementary material for: Estimation of chronic dietary intake of pesticide residues
Source: Rev Saude Publica. 2021 Jun 17;55:36. doi: 10.11606/s1518-8787.2021055002197 (PMC8225320; doi:10.11606/s1518-8787.2021055002197)
Supplement: Translation

# Translation: Estimativa de ingestão crônica de resíduos de agrotóxicos por meio da dieta

**Authors:** Jacqueline Mary Gerage Marques, Marina Vieira da Silva

**Affiliations:** I Brasil Universidade de São Paulo. Escola Superior de Agricultura Luiz de Queiroz. Programa de Pós-Graduação em ciência e tecnologia de alimentos. Piracicaba, SP, Brasil; II Brasil Universidade de São Paulo. Escola Superior de Agricultura Luiz de Queiroz. Departamento de Agroindústria, Alimentos e Nutrição. Piracicaba, SP, Brasil

**Keywords:** Contaminantes Químicos em Alimentos, Agroquímicos, efeitos adversos, Limite Máximo de Agrotóxico em Alimentos, Exposição a Praguicidas, Segurança Alimentar e Nutricional

## Abstract

**OBJETIVO:**

Estimar a ingestão diária máxima teórica dos agrotóxicos potencialmente consumidos, de forma crônica, pela população brasileira.

**MÉTODO:**

Utilizando os dados do bloco de consumo alimentar da Pesquisa de Orçamentos Familiares de 2008–2009 para caracterização da dieta da população, foi construído um banco de dados agrupando os alimentos com base na classificação NOVA. Considerando os valores de limite máximo de resíduos de cada agrotóxico autorizado no país até o ano de 2016, foram somados os limites de todos os alimentos consumidos, multiplicados pela quantidade consumida, gerando o índice de ingestão teórica máxima, que foi comparado com a ingestão diária aceitável.

**RESULTADOS:**

Os resultados mostram que dos 283 agrotóxicos considerados no banco de dados, 71 compostos tiveram estimativas de ingestão zero (25%), 144 compostos (50,8%) atingiram aos valores de ingestão diária aceitável e 68 compostos (24%) apresentaram mediana de ingestão que excedeu o valor diário aceitável. Quando realizada a estimativa de ingestão de agrotóxicos discriminando as distintas regiões do país, houve variação (entre 48 e 69 substâncias) na quantidade de compostos que excederam a ingestão diária aceitável devido aos diferentes padrões de consumo da população. As categorias dos produtos que mais excederam as estimativas são inseticidas, herbicidas e fungicidas.

**CONCLUSÃO:**

A aplicação dessa metodologia é válida para o primeiro passo na avaliação de risco, porém os valores resultantes podem ser diferentes da exposição real por não englobar outros fatores, como o uso combinado de agrotóxicos ou de produtos de uso não autorizado. É ressaltada a importância do desenvolvimento de pesquisas de dados específicos de consumo de alimentos nacionais de forma sistemática, gerando dados e análises que viabilizem uma avaliação pormenorizada sobre riscos.

## INTRODUÇÃO

A segurança alimentar e nutricional tem como fundamento inicial a relação entre oferta e consumo de alimentos por parte da população, considerando disponibilidade e acesso. Tem relação com diferentes áreas do conhecimento, como saúde, agricultura, economia, alimentação e meio ambiente, ganhando diferentes significados. É um conceito em evolução, se relacionando também com a sustentabilidade, que assume crescente importância em um cenário de mudanças climáticas, que impactam os preços e causam interrupções na cadeia de fornecimento de alimentos. A nutrição e soberania alimentar também contribuem para a evolução do conceito^1^.

A grande produção de *commodities* no país, impulsionada pelo aumento da demanda global de alimentos, aumentou em 135% o consumo total de agrotóxicos no período de 2000 a 2014^6^, potencializando assim a exposição aos resíduos desses produtos. Os resíduos de agrotóxicos podem ser uma substância ou a mistura de substâncias remanescentes nos alimentos ou no meio ambiente. Os resíduos também podem ser os produtos de conversão, degradação, e reação, assim como metabólitos e impurezas que tenham importância toxicológica^7^. As intoxicações ocorrem na forma aguda, quando há exposição a grande quantidade de resíduos do produto, ou crônica, quando há exposição a pequenas quantidades de forma prolongada. Nos alimentos, o potencial toxicológico dos resíduos ocorre não apenas em alimentos vegetais *in natura*, mas também em alimentos preparados (receitas caseiras) e processados, encontrados no mercado em geral.

São escassos os trabalhos que avaliam, em especial para a população brasileira, os riscos de intoxicação da população por meio da dieta. Essa avaliação embasa a identificação de substâncias potencialmente danosas, possibilitando a caracterização do risco. O conjunto de dados que viabiliza esse tipo de avaliação são os dados de consumo da população e dados antropométricos.

O objetivo do trabalho foi estimar a ingestão diária máxima teórica (IDMT) dos agrotóxicos potencialmente consumidos pela população brasileira, por meio da dieta, com base nos dados de consumo obtidos na Pesquisa de Orçamento Familiar (POF) conduzida pelo Instituto Brasileiro de Geografia e Estatística (IBGE) de 2008–2009, destacando as substâncias que extrapolaram os limites estabelecidos, de acordo com os produtos autorizados para uso no Brasil até o ano de 2016.

## MÉTODOS

O banco de dados utilizado para cálculo das estimativas foi construído utilizando os microdados do bloco de consumo alimentar da POF, que registra o consumo da população brasileira a partir de 10 anos de idade. Há disponibilidade de dados relativos a uma amostra de 13.569 domicílios, dos quais foram considerados 33.613 indivíduos para os quais havia identificação completa dos dados antropométricos, pois foi aplicado o peso corporal de cada indivíduo para as análises propostas.

No bloco de consumo alimentar da referida pesquisa, são registrados os itens alimentares em sua forma de consumo. Na construção do banco de dados, os alimentos foram agrupados de acordo com as culturas agrícolas que lhe dão origem, e assim foram aplicados os dados encontrados nas monografias dos agrotóxicos, quanto ao limite de resíduos^8^. Utilizando como base a classificação NOVA^9,10^, procedeu-se à classificação dos alimentos em três grupos: *in natura*, processados e preparados.

Foram considerados alimentos *in natura* os quais não se aplica nenhum tipo de preparação. Os alimentos preparados envolvem vários ingredientes e foram identificados em proporção, com base em receitas-padrão e tabelas de referência^11^. Quanto aos alimentos processados, foi pesquisada a composição dos alimentos em porcentagem de ingredientes, com base em rótulos de produtos, consulta às legislações vigentes da Agência Nacional de Vigilância Sanitária (Anvisa) ou Ministério de Agricultura, Pecuária e Abastecimento^15^, tabelas de referência^11^ e buscas na internet.

Também integraram o banco de dados os alimentos de origem animal, que foram agrupados em: carne bovina, carne suína, carne de frango, carne de aves, carne ovina, carne caprina, carne de mamíferos, miudezas de gado, miudezas de aves, ovos e leite. Para esses grupos foram utilizados os valores de limite máximo de resíduos (LMR) obtidos no *Codex Alimentarius*^23^, considerando apenas os compostos autorizados para uso no Brasil, visto que não temos publicação por parte da Anvisa cobrindo essa categoria de produtos.

Foram excluídos da pesquisa os alimentos e bebidas das categorias light, diet e orgânicos, bem como alimentos de composição não encontrada ou sem valor de LMR definido pela Anvisa. Resultaram, ao final, um total de 743 alimentos para análise de dados.

Foram adotados os valores de LMR registrados nas monografias dos agrotóxicos (n=283) com uso autorizado no país até o ano de 2016. Os valores de LMR foram somados de acordo com o registro de alimentos consumidos obtido da POF e multiplicados pela quantidade consumida, resultando no IDMT, que indica o grau de exposição, conforme a Equação 1.

**Equação 1.** Cálculo do IDMT^a^.

$$IDMT=\sum \left({LMR}_{i}\times C_{i}\right)$$

Os resultados foram comparados com os valores de ingestão diária aceitável (IDA), publicados nas monografias dos agrotóxicos pela Anvisa, conforme a Equação 2. Quando inexistentes os dados nacionais, foram considerados os valores de agências internacionais, tais como *Environmental Protection Agency* (EPA), *Codex Alimentarius* e do departamento de saúde do governo australiano^23^,considerando o menor valor quando havia mais de um registro para o mesmo composto.

**Equação 2.** Comparação IDTM com IDA.

$$\%IDA=\frac{IDMT\times 100}{\text{ IDA }\times \text{ peso corpóreo }}$$

## RESULTADOS

Dos 283 agrotóxicos estudados, 71 compostos (25%) tiveram estimativa de ingestão igual a zero. Identificou-se também que 144 compostos (50,8%) atenderam aos valores da IDA, e 113 deles tinham valor preconizado pela Anvisa. Os outros 68 compostos (24%) apresentaram mediana de ingestão que excedeu o valor da IDA, conforme dados apresentados na Tabela 1.

**Tabela 1:** Intervalos de extrapolação em relação à ingestão diária aceitável (IDA).

Intervalos de extrapolação em relação à IDA	Agrotóxico
(1~2) X > IDA (n = 32)	Acetamiprido, alacloro, aldicarbe, azoxistrobina, beta-cipermetrina, bifentrina, captana, carbosulfano, ciproconazol, ditiocarbamatos, edifenfós, esfenvalerato, famoxadona, fenoxaprope, fenpropatrina, fluasifope-p, hidrazida-maleica, iprodiona, malationa, mancozebe, molinato, novalurom, propargito, protioconazol, quinometionato, sulfentrazona, teflubenzurom, tetradiona, tiametoxam, tiofanato-metílico, tiram, triazofós.
(3~4) X > IDA (n = 18)	Cadusafós, carbendazim, cletodim, clormequate, clorpirifós, dimetoato, epoxiconazol, etofenproxi, fenamifós, fosmete, gama-cialotrina, haloxifope-p, iminoctadina, mevinfós, msma, paraquate, propinebe, protiofós.
(5~6) X > IDA (n = 6)	Carbaril, carbofurano, deltametrina, diafentiurom, pirimifos-metilico, tetraconazol.
(7~8) X > IDA (n = 2)	Dissulfotom, etiona.
(9~10) X > IDA (n = 3)	Diquate, diurom, propanil.
> 10 X IDA (n = 7)	Acefato, brometo de metila, diazinona, fentina, fipronil, fosfina, terbufos.

A Tabela 2 indica os 10 compostos com maiores estimativas de ingestão pela população, dos quais sete (acefato, brometo de metila, diazinona, fentina, fipronil, fosfina e terbufós) superaram dez vezes o valor da IDA. Os outros três (diquate, diurom e propanil) extrapolaram de nove a dez vezes o valor da IDA.

**Tabela 2:** Estimativa de agrotóxicos mais consumidos pela população brasileira por meio da dieta.

Agrotóxico	IDA (mg/kg)	Valor da mediana de consumo (mg/kg de peso corpóreo)	Classificação toxicológica
Brometo de metila	0,0004 (AU)	1,527778	I – extremamente tóxico
Fosfina	0,0003 (EPA)	0,007711	II – altamente tóxico
Fipronil	0,0002	0,004866	II – altamente tóxico
Acefato	0,0012	0,022826	III – medianamente tóxico
Diazinona	0,002	0,030805	II – altamente tóxico
Fentina	0,0005	0,006956	II – altamente tóxico
Terbufós	0,0002	0,002632	II – altamente tóxico
Diquate	0,002	0,020833	II – altamente tóxico
Diurom	0,002 (EPA)	0,020669	III – medianamente tóxico
Propanil	0,005 (EPA)	0,045147	III – medianamente tóxico

IDA: ingestão diária aceitável; AU: governo australiano; EPA: agência de proteção ambiental dos Estados Unidos da América.

Quando realizada a estimativa de ingestão de agrotóxicos distinguindo a região do país, houve variação na quantidade de compostos que excederam a IDA, devido à diferença no consumo e diversidade de alimentos condicionada por distintas variáveis, com destaque para rendimentos familiares, hábitos, identidade cultural da população e disponibilidade regional. A Tabela 3 indica a quantidade de compostos excedentes por região.

**Tabela 3:** Quantidade de compostos que excederam a ingestão diária aceitável de acordo com a região.

Região	Quantidade de agrotóxicos (compostos)
Norte	59
Nordeste	62
Sudeste	69
Sul	48
Centro-Oeste	69

Na região Norte, foram observados os mesmos dez compostos com maiores medianas de ingestão obtidos para toda a população brasileira, mantendo em primeiro lugar o composto brometo de metila, com diferenças na quantidade de consumo dos outros compostos. O propanil, produto com poucos registros de intoxicação e indicado para aplicação na cultura de arroz^26^, ocupou o segundo lugar.

O composto dissulfotom excedeu o limite da IDA na região Nordeste, o que não ocorreu em nenhuma outra região. É um inseticida organofosforado, com aplicação na cultura de café, o segundo alimento com maior número de referências no registro alimentar do primeiro dia da POF 2008–2009, que apresenta o maior consumo *per capita* do país nessa região, alcançando 230,4 g/dia^26^.

Para a região Sudeste, foi identificado o composto etiona entre os dez compostos com maior potencial de ingestão. Esse é um composto com indicação aplicação foliar nas culturas de abacaxi, algodão, berinjela, café, citros, maçã, melancia, melão, pera, pimentão e tomate^26^, englobando alimentos comumente consumidos nos estados da região.

Em análise específica, utilizaram-se apenas os dados relativos ao estado de São Paulo, pois suas características socioeconômicas se diferenciam dos demais estados e regiões, sendo consideradas informações relativas a 2.250 indivíduos. Para esse estado, ultrapassaram os limites determinados pela IDA os compostos etiona e propamocarbe. Pesquisas mostram que ambos apresentaram redução nos resíduos pelos procedimentos de processamento, como refino de óleo, lavagem e cocção^29,30^. Porém, embora o processamento dos alimentos possa reduzir a carga de resíduos, não é possível garantir que atinjam o nível da IDA.

A região Sul se destaca por apresentar menor quantidade de compostos que excederam o limite da IDA. Segundo os dados da Vigitel^31^, as capitais dos estados da região Sul estão entre as dez cidades do país cuja população mais consome frutas e hortaliças, sendo o melhor índice identificado em Florianópolis (Santa Catarina). Quanto ao uso dos agrotóxicos, os estados com maior registro de consumo são o Paraná com 11,6% e Rio Grande do Sul com um consumo médio de 10,2% do total aplicado no país^32^. Entretanto, essa foi a região que registrou maior consumo de alimentos orgânicos, de acordo com a POF.

Quanto ao Centro-Oeste, o perfil dos compostos que excederam os limites da IDA foi semelhante ao do estado de São Paulo, com destaque também para o composto etiona.

## DISCUSSÃO

A exposição crônica aos resíduos dos agrotóxicos organofosforados e carbamatos, categorias em que a maior parte dos agrotóxicos que excederam a IDA se enquadram, pode se relacionar com uma série de sintomas, como efeitos neurotóxicos, alterações cromossômicas, lesões hepáticas e renais, arritmias, alergias, asma, doença de Parkinson, cânceres e perda auditiva^33^. Os produtos dessas categorias podem ainda atuar como inibidores de acetilcolinesterase, o que afeta a transmissão dos impulsos nervosos ao cérebro.

O brometo de metila, que se destacou como o produto com maior potencial de ingestão, é utilizado na aplicação em caixas de madeira que acondicionam os produtos durante o armazenamento pós-colheita. Esse composto apresenta um elevado LMR, porém com uma IDA baixa, por isso figura com um valor alto de IDTM, chamando a atenção pelo potencial de alta ingestão. Também é importante notar que, em geral, os herbicidas, uma das categorias mais comercializada no mundo, possuem elevada IDA em relação aos fungicidas e inseticidas, e por isso não se configuraram uma categoria de destaque nos resultados.

Esse tipo de avaliação é válido para o primeiro passo na avaliação de risco, pois o LMR não é o melhor indicador para os cálculos de níveis médios de resíduos. Realizar a soma da totalidade das doses de agrotóxicos também não é o ideal para a avaliação de risco cumulativa, devendo ser estudados os efeitos cumulativos e sinérgicos das substâncias que causam os mesmos efeitos tóxicos em tecidos, órgãos e sistemas fisiológicos e podem produzir toxicidade conjunta e cumulativa – mesmo que não tenham modos de ação semelhantes^34^.

Cabe destacar que essa estimativa não considera o volume de pesticidas adquirido e utilizado de forma ilegal, que invariavelmente entram no país sem permissão, ou ainda o uso de produtos falsificados, que não tiveram a eficácia e segurança do produto atestada pelos órgãos responsáveis. O comércio dos agrotóxicos ilegais corresponde a 24% do mercado de defensivos agrícolas do Brasil, segundo dados da Federação das Indústrias do Estado de São Paulo^38^.

Como estratégia para a gestão de segurança alimentar do país, é importante que haja um programa eficiente de monitoramento de resíduos e do sistema de aplicação de agrotóxicos realizado continuamente. Esses resultados poderão embasar medidas, tais como: educação no campo para a aplicação das boas práticas agrícolas, avaliação de risco à saúde pela exposição e reavaliação de produtos e substâncias.

Outro ponto de destaque é a utilização de dados individuais de consumo de alimentos no banco de dados. É importante para esse tipo de estimativa a utilização de amostras de grupos populacionais de forma proporcional, apresentando a estratificação por faixa etária, sexo e educação.

O estudo de Meira et al.^39^ reúne estimativas de consumo de agrotóxicos, tendo como foco o grupo de crianças em idade escolar da rede pública de ensino de um município do interior do estado de São Paulo. Os autores mostram que, dos 272 compostos considerados no estudo, 58 superaram o valor máximo de ingestão. Alguns desses compostos podem atuar como desreguladores endócrinos, podendo acarretar alterações no desenvolvimento, uma preocupação fundamental para a amostra em questão.

Com isso se evidencia a necessidade do incentivo ao desenvolvimento de estudos nacionais específicos sobre fatores de processamento para os níveis de resíduos encontrados. Os estudos disponíveis demonstram que todos os tipos de processamento contribuem para a redução de risco pela exposição^40^

Uma pesquisa em uma comunidade de vegetarianos em Israel^48^identificou aumento da concentração de resíduos de organofosforados em amostras de urina, e os moradores que consumiam maior parcela de alimentos orgânicos apresentaram resultados menores de resíduos dos agrotóxicos. Com isso o consumo de produtos orgânicos pode oferecer alguma proteção contra a exposição. A agroecologia é uma forma de manejo sustentável da produção agrícola, considerando questões sociais, políticas, culturais, ambientais e éticas – condição de trabalho no campo, compatibilidade da cultura ao ecossistema e o nível de industrialização de todo o processo. É uma modalidade que evita a utilização de agrotóxicos e fertilizantes químicos e estimula o plantio de orgânicos, sendo uma categoria impulsionada pela agricultura familiar. Porém devem ser considerados os resultados da produção em escala, os custos envolvidos e tempo destinado ao plantio e colheita, assim como a produtividade.

As perspectivas de uso futuro de agrotóxicos devem ser avaliadas, não apenas quanto aos efeitos sobre as culturas, ocorrência de pragas e eficiência dos pesticidas, mas também suas implicações para o desenvolvimento tecnológico dos regulamentos e situação econômica. Com a expectativa das mudanças climáticas, o aumento das temperaturas atuará sobre a mudança nos regimes de chuvas, favorecendo o desenvolvimento de pragas e patógenos. Como consequência prevê-se o aumento do uso de agrotóxicos em dosagem, frequência de aplicação e tipos de produtos. Portanto, devem surgir novos compostos, ampliação das aplicações combinadas e aumento na toxicidade dos ativos, assim elevando a exposição aos resíduos no consumo de alimentos^49^.

É importante destacar que existem poucos estudos sobre os efeitos ecotoxicológicos decorrentes da aplicação de agrotóxicos em regiões tropicais, sendo majoritariamente utilizados como referências estudos aplicados em regiões temperadas. É indicada a adaptação dos testes existentes para as regiões tropicais^47^.

## CONCLUSÃO

A garantia de alimentos livres de resíduos integra o direito humano à alimentação adequada, reforçando a necessidade de rigoroso monitoramento de resíduos de agrotóxicos, considerado importante para um programa nacional de segurança alimentar e nutricional. De acordo com os resultados obtidos, existe uma série de agrotóxicos aplicados nas culturas consumidas no Brasil com potencial de ingestão que podem exceder aos índices preconizados. Assim, sugere-se ao sistema de saúde nacional avaliar o potencial real de exposição aos resíduos de agrotóxicos, considerando ainda que estamos diante de perspectivas de mudanças globais em relação ao clima, o que implicará mudanças no uso desses produtos.

Se faz necessária também a geração sistemática de dados de consumo de alimentos para a avaliação de riscos crônicos, agudos e cumulativos de forma mais realista. Não há forma de avaliação dos efeitos da exposição por meio do consumo de alimentos e água, sendo possível apenas a utilização de modelos com base no princípio da precaução; entretanto, já se sabe que a exposição a baixas doses induz à morte celular e redução de viabilidade das células.

## Footnotes

a World Health Organization. Guidelines for predicting dietary intake of pesticide residues (revised): prepared by the Global Environment Monitoring System – Food Contamination Monitoring and Assessment Programme (GEMS/Food) in collaboration with the Codex Comitee on Pesticide Residues. Zurich, 1997, 40 p.
